# Whole Genome Assembly of Human Papillomavirus by Nanopore Long-Read Sequencing

**DOI:** 10.3389/fgene.2021.798608

**Published:** 2022-01-04

**Authors:** Shuaibing Yang, Qianqian Zhao, Lihua Tang, Zejia Chen, Zhaoting Wu, Kaixin Li, Ruoru Lin, Yang Chen, Danlin Ou, Li Zhou, Jianzhen Xu, Qingsong Qin

**Affiliations:** ^1^ Laboratory of Human Virology and Oncology, Shantou University Medical College, Shantou, China; ^2^ Computational Systems Biology Lab, Department of Bioinformatics, Shantou University Medical College, Shantou, China; ^3^ Department of Gynecologic Oncology, Cancer Hospital of Shantou University Medical College, Shantou, China; ^4^ Undergraduate Program of Innovation and Entrepreneurship, Shantou University Medical College, Shantou, China; ^5^ Guangdong Provincial Key Laboratory of Infectious Diseases and Molecular Immunopathology, Shantou, China; ^6^ Guangdong Provincial Key Laboratory for Diagnosis and Treatment of Breast Cancer, Shantou, China

**Keywords:** HPV, nanopore sequencing, cervical cancer, integration, episomal genome

## Abstract

Human papillomavirus (HPV) is a causal agent for most cervical cancers. The physical status of the HPV genome in these cancers could be episomal, integrated, or both. HPV integration could serve as a biomarker for clinical diagnosis, treatment, and prognosis. Although whole-genome sequencing by next-generation sequencing (NGS) technologies, such as the Illumina sequencing platform, have been used for detecting integrated HPV genome in cervical cancer, it faces challenges of analyzing long repeats and translocated sequences. In contrast, Oxford nanopore sequencing technology can generate ultra-long reads, which could be a very useful tool for determining HPV genome sequence and its physical status in cervical cancer. As a proof of concept, in this study, we completed whole genome sequencing from a cervical cancer tissue and a CaSki cell line with Oxford Nanopore Technologies. From the cervical cancer tissue, a 7,894 bp-long HPV35 genomic sequence was assembled from 678 reads at 97-fold coverage of HPV genome, sharing 99.96% identity with the HPV sequence obtained by Sanger sequencing. A 7904 bp-long HPV16 genomic sequence was assembled from data generated from the CaSki cell line at 3857-fold coverage, sharing 99.99% identity with the reference genome (NCBI: U89348). Intriguingly, long reads generated by nanopore sequencing directly revealed chimeric cellular–viral sequences and concatemeric genomic sequences, leading to the discovery of 448 unique integration breakpoints in the CaSki cell line and 60 breakpoints in the cervical cancer sample. Taken together, nanopore sequencing is a unique tool to identify HPV sequences and would shed light on the physical status of HPV genome in its associated cancers.

## Introduction

Human papillomavirus (HPV), a double-stranded circular DNA virus, is a causal agent for most cervical cancers (Zur Hausen, 2002; Abu-Lubad et al., 2020) and is also associated with anal cancer (Alemany et al., 2015), oropharyngeal cancer (Meng et al., 2020), and vaginal cancer (Hellman et al., 2004). Until now, more than 200 genotypes of HPV have been identified (Bzhalava et al., 2015) and can be classified as high-risk or low-risk genotypes based on their properties of tumorigenesis (de Villiers, 2013). Persistent infection by high-risk HPV is more likely to initially lead to precancerous cervical intraepithelial neoplasia (CIN) and finally result in invasive cervical carcinoma (ICC) (Walboomers et al., 1999; Zur Hausen, 2002; Woodman et al., 2007; Yuan et al., 2021). Integration of HPV DNA into the host genome is considered a key event in driving cervical carcinogenesis (Pett and Coleman, 2007; Zhang et al., 2016; Warburton et al., 2018). The break sites in the cellular–viral junction region are commonly found in E1 and E2 genes in the viral genome, which leads to the loss of expression of viral E2 protein and the increased expression of viral oncoproteins (E6 and E7) that inhibit cell cycle checkpoint proteins p53 and pRB (Woodman et al., 2007; Groves and Coleman, 2015) and is essential for the proliferation and survival of HPV-related cancer cells (Hoppe-Seyler et al., 2018). The frequency of HPV integration is positively correlated with cervical intraepithelial neoplasia (CIN) (Hudelist et al., 2004; Guo et al., 2007; Wang et al., 2013). In HPV-related cancers, the physical status of HPV genome has been found to be episomal, integrated, or mixed (Park et al., 1997; Nambaru et al., 2009; Niya et al., 2019). For example, the CaSki cells, a naturally derived cervical carcinoma cell line, contain a high number of concatemeric HPV genomic sequences inserted within cellular genome (Yee et al., 1985) and focal genomic variations at the integration locus (McBride and Warburton, 2017). It was previously reported that patients with episomal HPV in cancer cells have a better survival rate than those with an integrated HPV genome (Nambaru et al., 2009; Kiseleva et al., 2013). Various methods have been used to detect the physical status of the HPV genome, such as PCR-based methods (Carmody et al., 1996; Luft et al., 2001), fluorescence *in situ* hybridization (FISH) (Triglia et al., 2009; Houldsworth, 2014; Olthof et al., 2015), whole genome DNA sequencing (WGS) and high-throughput viral integration detection (HIVID) (Hu et al., 2015), RNA sequencing (RNA-seq) (Klaes et al., 1999), and single-molecule sequencing technology (SMRT-seq) (Yu et al., 2021). However, each method has its strengths and limitations. For example, whole genome sequencing by next-generation sequencing (NGS) technologies (Hu et al., 2015; Kamal et al., 2021) requires a high coverage of human genome ( >30 x coverage) and a complicated algorithm to analyze HPV integrations in human genome. Short-reads (100–500 bp) generated by NGS can lead to errors and ambiguity in mapping viral integration and assembling repetitive sequences (Alkan et al., 2011; Treangen and Salzberg, 2011).

Nanopore long-read sequencing is a third-generation sequencing technology released by Oxford Nanopore Technologies in 2014 (Jain et al., 2015), which determines DNA bases by reading the current fluctuation of passing nucleotides through biological nanopores (Lu et al., 2016) and therefore has the ability of reading ultra-long sequences (Jain et al., 2018). Ultra-long reads are more likely to cover the complete viral genome, highly repetitive regions, and structural variations in human genome, allowing continuous and complete genome assembly (Jain et al., 2018; Senol Cali et al., 2018). With their long reading capacity, nanopore sequencing technologies have shown great advantages in the rapid surveillance of Ebola (Hoenen, 2016; Quick et al., 2016) and Zika viruses (Quick et al., 2017), genotyping and genetic diversity analysis of hepatitis B virus (HBV) (Sauvage et al., 2018; McNaughton et al., 2019; Astbury et al., 2020), and monitoring emerging infectious disease outbreaks (Zhu et al., 2020). Whole genome sequencing (WGS) of SARS-CoV-2 with nanopore sequencing technologies provided a unique tool to analyze viral transmission, evolution, and genomic variation (Bull et al., 2020; Lin et al., 2021). In this study, as a proof of concept, we sequenced total DNA extracted from a cervical cancer tissue and a CaSki cell line with nanopore sequencing technologies, assembled HPV genomes with several bioinformatic tools, and analyzed the physical status of HPV.

## Materials and Methods

### Sample Collection

A fresh tissue sample was obtained from a 74-year-old patient with cervical carcinoma without prior chemotherapy or radiotherapy at the Cancer Hospital of Shantou University Medical College (Guangdong, China) in 2019. An informed consent form was obtained from the patient. This study was approved by the ethical review board of Shantou University Medical College (approval number: SUMC-2020-51).

### Cell Culture

CaSki cells (CRL-1550, ATCC, United States) were cultured in RPMI-1640 medium (SH30809.01B, Hyclone, Logan, UT, United States) with 10% fetal bovine serum (Ausbian, Austrilia), 100 U/ml penicillin, and 100 mg/ml streptomycin in a humidified incubator with 5% CO_2_ at 37°C. C-33A cells (TCHu176, Shanghai Cell Bank, Chinese Academy of Sciences, China) were cultured in Dulbecco’s modified Eagle medium (SH30022.01, Hyclone, Logan, UT, United States) containing 10% fetal bovine serum at 37°C with 5% CO_2._

### MinION DNA Library Preparation

Total DNAs were respectively extracted from a cervical cancer tissue sample and CaSki cells using a QIAamp^®^ DNA Mini Kit (C51304, Qiagen, Hilden, Germany) according to the manufacturer’s protocol and quantified using a Qubit dsDNA HS Assay Kit (Q33230, Thermo Fisher Scientific, Waltham, MA, United States). Two μg of DNA fragments ( >10 kb) was used for library preparation and sequenced using a MinION SQK-LSK109 Oxford nanopore sequencing kit (SQK-LSKSP9, Oxford Nanopore Technologies, Oxford, United Kingdom) according to the manufacturer’s instructions. Briefly, DNA was repaired with FFPE DNA Repair Mix and End repair/dA-tailing Module reagents [E7695, New England BioLabs (NEB), Ipswich, MA] and purified with AMPure XP beads (A63880, Beckman Coulter, United States), washed with 70% ethanol, and eluted with nuclease-free water. Sequencing adapters were added to the 3′ ends of fragmented DNA using Adapter Mix and Quick T4 DNA Ligase with Ligation Buffer (NEB) and purified with AMPure beads. The prepared library was added into a SpotON flow cell (FLO-MIN106D, Oxford Nanopore Technologies) and sequenced using a MinION sequencer Mk1B.

### *De Novo* Assembly of HPV Genome With Bioinformatic Tools

**FIGURE 1:**
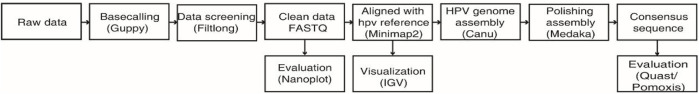
Workflow chart of bioinformatic analysis and *de novo* assembly of the HPV genome from nanopore sequencing reads.

As shown in Figure 1, FAST5 files generated by the MinION sequencing device were converted to FASTQ using GUPPY (v3.1.5), provided in the MinKNOW software package**.** Low-quality reads (under a Q-score = 7 threshold) were removed using Filtlong (v0.2.0). The distributions and read lengths of data were evaluated using NanoPlot (https://github.com/wdecoster/NanoPlot). For the assembly of HPV genome, sequences were aligned to a HPV reference sequence downloaded from the Papillomavirus Episteme database (PaVE) (https://pave.niaid.nih.gov/) with Minimap2 (v2.17) using the following parameters (-t 20 -ax map-ont -Y) and extracted using SAMtools (v1.9) software (Li, 2018). The coverage and sequencing depth were calculated using Bamdst (v1.0.9) (https://github.com/shiquan/bamdst) and the mapping of genomic data was visualized using Integrative Genomic Viewer (IGV) (Thorvaldsdóttir et al., 2013). Next, the assembly of the HPV genome was done using Canu (Koren et al., 2017) under the following parameters (genome Size = 8k -stop on Low Coverage = 5 corrected Error Rate = 0.105 -nanopore-raw). Furthermore, the assembled HPV genome was corrected by using a polishing software called medaka (v1.2.1) and with the medaka consensus option (GitHub - nanoporetech/medaka: Sequence correction provided by ONT Research). A consensus HPV genomic sequence was then obtained by removing the duplicate parts, followed by assessing the assembly quality using Quast (Gurevich et al., 2013). We evaluated the base level error rate of the assembly result using Pomoxis software developed by Oxford Nanopore Technologies (https://github.com/nanoporetech/pomoxis). Finally, we used the dnadiff option of mummer (4.0.0) (Marçais et al., 2018) software to analyze the differences between assembled sequences and HPV reference genomes deposited in PaVE.

### Analysis of HPV Integration Sites

Reads that are both aligned to human reference genome (UCSC, hg38) and viral reference genome are defined as chimeric sequences, which are screened using Minimap2 software. Chimeric sequences were extracted using SAMtools (v1.9). The cellular–viral joint sites (also known as breakpoints) were further determined using BLAT (v35) (Kent, 2002) by repositioning chimeric sequences to human and HPV genome using BLAT (v35) with the following parameters (-stepSize = 5 -repMatch = 2,253 -minScore = 20 -minIdentity = 0). HPV integration sites were defined as gaps or overlaps between host and viral alignments resulting in less than 10 bp. Those with the highest score were selected as credible positions. Gene information of the breakpoint sites was annotated using the bedtools intersect option based on files downloaded from the website (https://www.gencodegenes.org/).

### PCR Verification

To verify the HPV35 genome obtained by nanopore sequencing, 17 pairs of primers (Table 1) were designed to amplify HPV genome from the cervical cancer tissue to ensure the full coverage of an intact HPV genome. Each pair of primers was designed to amplify about 400–600 bp using a PCR Mix kit (C113, Vazyme, Nanjing, China). PCR products were purified by electrophoresis and sequenced using the Sanger sequencing technologies. To verify HPV integration sites, two primers **(** Table 1 **)** flanking integration sites were designed to amplify cellular–viral joint regions, and amplified products were subjected to gel purification and Sanger sequencing.

**TABLE 1:** Primers used in this study.

Target	Primer designation	Sequence
HPV35: 212–766	Primer1(F)	5′-CAA​GAA​TTA​CAG​CGG​AGT​GAG​GT-3′
	Primer1(R)	5′-ACA​GAC​GTA​GTG​TCG​CCT​CAC​AT-3′
HPV35: 609–1,165	Primer2(F)	5′-ACC​CGA​GGC​AAC​TGA​CCT​ATA​CTG​T-3′
	Primer2(R)	5′-AGC​TCA​CGC​TGC​TAA​GTG​GAC​TA-3′
HPV35: 1,139–1756	Primer3(F)	5′-CTA​GTA​GTC​CAC​TTA​GCA​GCG​TGA​G-3′
	Primer3(R)	5′-TTG​GTG​GTT​GTA​TTA​GCA​TAC​TTG​C-3′
HPV35: 1,426–2,140	Primer4(F)	5′-AGT​AAT​GCA​AAC​GCA​GCT​ATG​TTG-3′
	Primer4(R)	5′-AGT​CAC​CGT​CAT​CGT​CCA​CCT​TT-3′
HPV35: 2,124–2,708	Primer5(F)	5′-GGA​CGA​TGA​CGG​TGA​CTG​GAG​G-3′
	Primer5(R)	5′-TTG​TCC​TCT​TCC​TCG​TGC​AAA​T-3′
HPV35: 2,558–3,086	Primer6(F)	5′-ACT​TAC​ATA​GCA​GGG​TAG​TGG​TCT​T-3′
	Primer6(R)	5′-CAC​CAT​CAA​ATT​GTA​CTT​CCA​CTG​T-3′
HPV35: 2,888–3,405	Primer7(F)	5′-GTG​GTT​CCA​ACG​CAG​GCC​ATT​TC-3′
	Primer7(R)	5′-CCC​ACG​GAG​CAG​GCT​TTG​GTA​TG-3′
HPV35: 3,377–3,910	Primer8(F)	5′-GAG​ACC​CAT​ACC​AAA​GCC​TGC​TC-3′
	Primer8(R)	5′-GCA​ATA​GCG​AAC​GTA​CAA​GCA​GA-3′
HPV35: 3,885–4,306	Primer9(F)	5′-GTG​TCT​GCT​TGT​ACG​TTC​GCT​AT-3′
	Primer9(R)	5′-AAC​ATC​TGG​TGG​ACA​AGT​TCC​TG-3′
HPV35: 4,239–4,870	Primer10(F)	5′-TTA​AAC​GTG​CAT​CTG​CAA​CAC​AAC​T-3′
	Primer10(R)	5′-AGG​GCG​AGA​CCC​TGG​AAT​AGG​C-3′
HPV35: 4,585–5,186	Primer11(F)	5′-TGT​TAC​ACC​AAG​GGT​CCC​ACC​TA-3′
	Primer11(R)	5′-CCT​GAT​AAT​AAT​GTA​CCC​GTG​CC-3′
HPV35: 5,075–5,640	Primer12(F)	5′-CCT​GCA​CTA​ACA​TCT​AGG​AAA​GG-3′
	Primer12(R)	5′-GCA​GGT​AGA​CAG​TGG​CTT​CGT​TA-3′
HPV35: 5,426–5,945	Primer13(F)	5′-CCT​ATA​ACA​GCA​GGG​CCA​GAC​AT-3′
	Primer13(R)	5′-TAC​ACC​CAA​TGG​CTG​ACC​ACG​AC-3′
HPV35: 5,874–6,505	Primer14(F)	5′-GAT​CCT​GCC​TCC​CAG​CGT​TTG​GT-3′
	Primer14(R)	5′-GCA​TCG​GAG​GTT​ACC​ATA​GAG​CCA​C-3′
HPV35: 6,477–7,057	Primer15(F)	5′-CCT​AGT​GGC​TCT​ATG​GTA​ACC​TCC​G-3′
	Primer15(R)	5′-GGA​GCT​GCA​CGC​TTG​CCT​AAT​CT-3′
HPV35: 6,964–7,627	Primer16(F)	5′-CAG​ACT​TAG​ATC​AAT​TTC​CGT​TGG-3′
	Primer16(R)	5′-GTG​TGG​GTG​GAC​CAC​AAG​TAT​GAA-3′
HPV35: 7,603–233	Primer17(F)	5′-TTT​CAT​ACT​TGT​GGT​CCA​CCC​A-3′
	Primer17(R)	5′-CTC​ACT​CCG​CTG​TAA​TTC​TTG​TT-3′
Divergent primers	Primer18(F)	5′- ACA​GCC​TGT​GAT​GTT​ACA​TAG​CG -3′
	Primer18(R)	5′- CAG​ACT​TAG​ATC​AAT​TTC​CGT​TGG -3′
HPV35: E2	primer(F)	5′-TAT​GGG​AAG​TGC​ATG​TGG​GTG​GTC-3′
	primer(R)	5′-GCA​CTG​AGT​CGC​ACT​CGC​TTG​G-3′
HPV35: E6	primer(F)	5′-CTG​AAC​GAC​CTT​ACA​AAC​TGC-3′
	primer(R)	5′-TCA​CTC​CGC​TGT​AAT​TCT​TGT-3′
HPV16: E2	primer(F)	5′-CAT​CAG​TAA​CTG​TGG​TAG​AGG​GTC-3′
	primer(R)	5′-GGA​TAC​TTC​GTT​GCT​GCT​AAA​C-3′
HPV16: E6	primer(F)	5′-GAG​CGA​CCC​AGA​AAG​TTA​CCA​C-3′
	primer(R)	5′-ACG​TCG​CAG​TAA​CTG​TTG​CTT​G-3′
β-actin	primer(F)	5′-TCC​TCC​TGA​GCG​CAA​GTA​CTC-3′
	primer(R)	5′-CGG​ACT​CGT​CAT​ACT​CCT​GCT​T-3′
PRR30 (BP)	primer(F)	5′-GCT​TTC​CCT​CAA​CTA​CTG​CCC​TGT​G-3′
	primer(R)	5′-ATG​CGC​CAA​CGC​CTT​ACA​TAC​CG-3′

### Plasmid Preparation

Partial E2 and E6 genes of HPV35 were amplified from DNA extracted from the cervical cancer tissue by primers 7 (F, R) and primers 1 (F, R) in Table 1 and cloned into the pMD19-T vector (6,013, Takara, Dalian, China). Plasmids were amplified in DH5α cells according to the manufacturer’s instructions (KTSM101L, KT Life Technology, Shenzhen, China) and isolated using a Plasmid Mini Kit (P1105, GBCBIO Technologies, Guangzhou, China).

### Determine Physical Status of HPV Genome in Cervical Cancer Tissue by Long-Range PCR and qPCR

To determine the existence of a circular HPV genome in the cervical cancer tissue and CaSki cells (no episomal HPV) (Yee et al., 1985), the extracted DNAs were treated with or without exonuclease V (M0345S, NEB) at 37°C for 3 h. A pair of divergent primers (primer 18 F, R) located in the L1 region of HPV35 (Table 1) was used to amplify a circular HPV35 genome. The circular DNA is resistant to exonuclease V. In addition, copy numbers of E2 and E6 in cervical cancer tissue and CaSki cells were quantified by qPCR using TB green qPCR kits (RR420A, Takara, Dalian, China) and calculated based on a standard curve established using 10-fold serially diluted E2 or E6 plasmids. The primers for E2 and E6 are listed in Table 1. The resistance of E2 or E6 to exonuclease V digestion was calculated based on qPCR results.

## Results

### Assembly of HPV Genome From Data Generated by the MinION Nanopore Sequencing Platform

**FIGURE 2:**
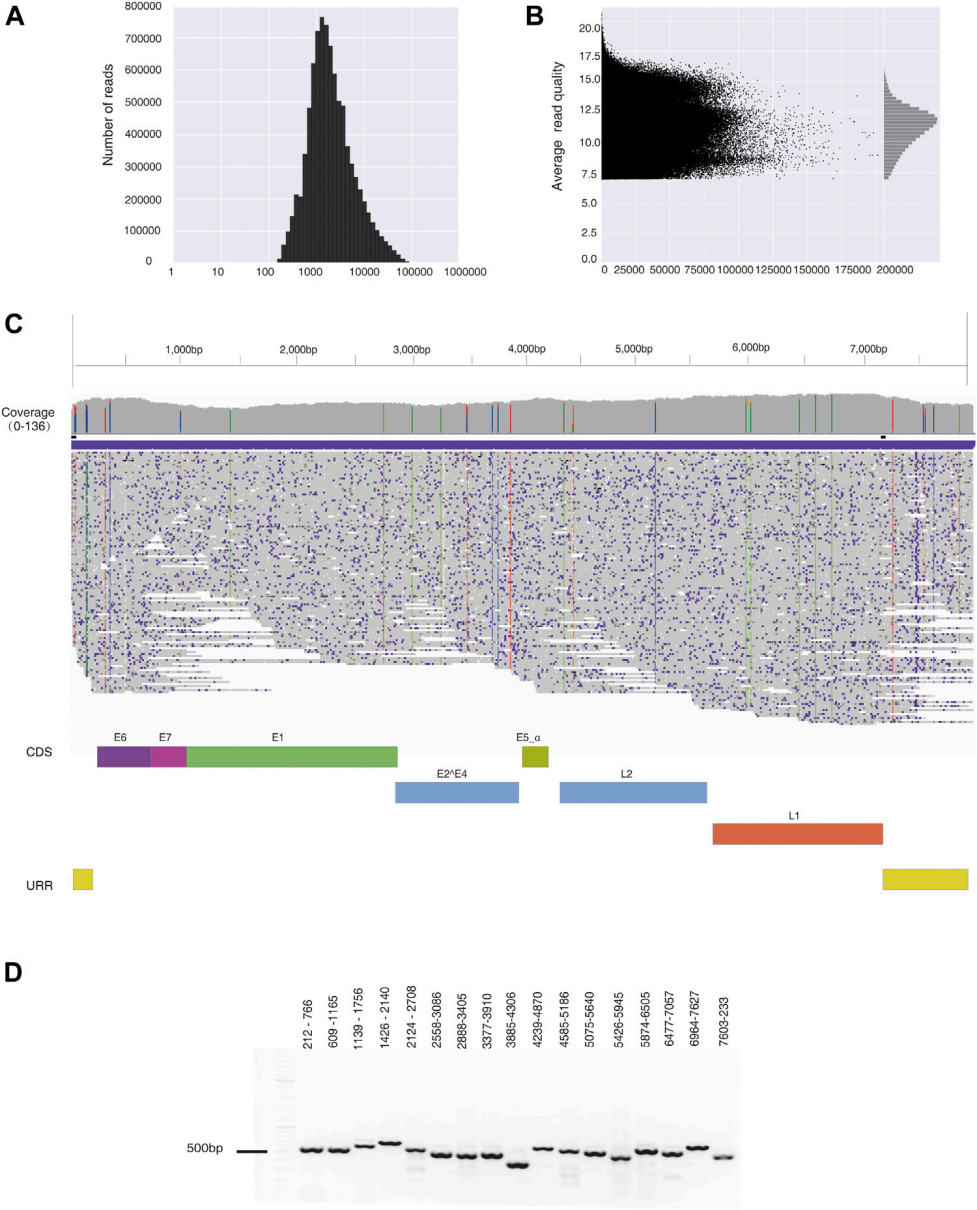
HPV35 genome was assembled from data generated by nanopore sequencing and further amplified by PCR. **(A)** The distribution of reads generated by nanopore sequencing from a cervical cancer tissue sample. **(B)** The distribution of the read quality of nanopore sequencing data. **(C)** *De novo* assembly of the HPV35 genome from 678 reads generated by nanopore sequencing, which is shown in the Integrative Genomics Viewer (IGV). **(D)** Seventeen HPV35 genomic segments were amplified from DNA extracted from a cervical cancer tissue sample.

After filtering data with low-quality scores (Q < 7), 8,683,345 reads were obtained from a cervical cancer tissue sample. The average read length (N50) equals 9,875 bp (Figure 2A), and the mean read quality score is 11.2 (Figure 2B). A total of 36 Gb of data ensured 11.6-fold coverage of the human genome. Out of these, 678 reads are aligned to HPV35 genome (PaVE, GI:396997), accounting for 0.01% of total reads. The average sequencing depth for the assembled HPV35 genomic DNA is 97 x. A final consensus HPV sequence is 7,894 bp-long (Figure 2C, Supplementary Table S3), which shares 99.93% identity with a reference sequence (HPV35 16B, NCBI: KX514416.1). To further validate the assembled HPV35 from nanopore sequencing, 17 segments that cover the whole HPV genome were amplified from the cervical cancer tissue sample and sequenced with Sanger sequencing (Figure 2D). The final assembled genome from Sanger sequencing is 7,894 bp (Supplementary Table S3), which is 99.96% identical to the sequence obtained by nanopore sequencing. In contrast, 4,211,267 reads were obtained from CaSki cells after filtering low-quality reads (Q score <7). The average read length (N50) is 11,862 bp, and the mean read quality score is 10.6. A total of 30 Gb data ensured about 10-fold coverage of the human genome. 15,947 reads were aligned to HPV16 genome (NCBI: U89348), accounting for 0.38% of total reads, and the average sequencing depth for HPV genome was 3,857 x. The final consensus HPV sequence is 7904 bp-long (Supplementary Table S3), which shares 99.99% identity with the reference sequence (NCBI: U89348). Taken together, from the whole genome sequencing data generated by nanopore sequence technology, the assembly of HPV genome with high accuracy can be achieved with our bioinformatic strategy.

### Unique Features of Ultra-Long Reads Generated by Nanopore Sequencing Reveal Different Forms of HPV Genome in Cervical Cancer Tissue Sample and CaSki Cells

**FIGURE 3:**
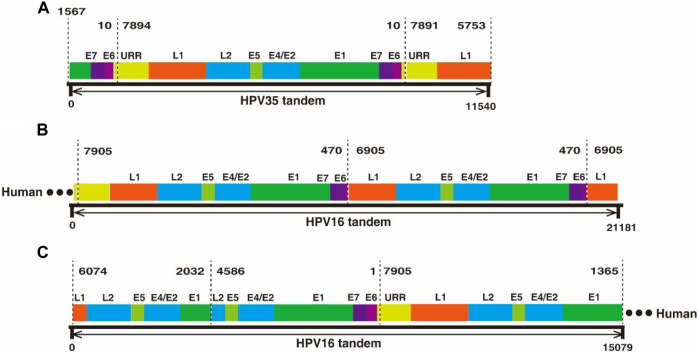
Nanopore sequencing found tandem HPV genomes in cervical cancer tissues and CaSki cells, respectively. **(A)** An 11.54 kb-long HPV 35 tandem genomic sequence was obtained by nanopore sequencing. The 11.54 kb-long sequence was aligned to HPV35 16B (NCBI: KX514416.1), which consists of three genomic segments, E1-E7-E6 (1567–10 nt), a whole genome frame (7894–10 nt), and URR-L1 (1561–10 nt. **(B)** A 21.18 kb-long HPV tandem sequence flanked with human genomic sequence at one end was obtained by nanopore sequencing from CaSki cells. The sequence was aligned to HPV16 (NCBI: U89348), in which a truncated genome (470–7905 nt) was connected to another truncated one (470–6905 nt) in a head-to-tail manner. **(C)** Another 15.07 kb-long HPV tandem sequence was obtained by nanopore sequencing from CaSki cells and was flanked at one end by the human gene; concatemers are formed by joining of incomplete HPV genomes with two spliced sites at 2032 nt and 4,586 nt.

In the two datasets generated from cervical cancer tissue and CaSki cells, there are two types of ultra-long reads containing viral sequences, partial concatemeric amplicons, and chimeric cellular–viral sequences as showed in Figure 3. For example, an 11.54 kb-long HPV nucleotide sequence (Supplementary Table S3) obtained from the cervical cancer sample consists of a nearly intact HPV genome (7,894 bp) flanked by two adjacent ends of HPV genome (Figure 3A), which could be derived from the breakage of concatemeric amplicons during the rolling circle replication of HPV genome or from integrated tandem viral genomic sequences as reported in CaSki cells (Yee et al., 1985; Mincheva et al., 1987). Interestingly, in this study, 300 tandem viral genomic sequences flanked with cellular genes were found in CaSki cells. Representative features of the sequences are drawn as shown in Figures 3B,C and sequences are attached in Supplementary Table S3. Most of the tandem viral genomic sequences are composed of incomplete genomic units joined at 4 hot spliced sites (position 470 nt (E6), 6,905 nt (L1), 2032 nt (E1), and 4,586 nt (L2)), which is consistent with previous studies (Meissner, 1999; Xu et al., 2015). In total, 60 and 448 chimeric cellular-viral reads (Supplementary Tables S1, S2) were found in the cervical cancer tissue and CaSki cells, respectively, which would shed light on hot spots of HPV integration in human genome.

### Determination of Physical Status of HPV Genome in a Cervical Cancer Tissue Sample and CaSki Cells

**FIGURE 4:**
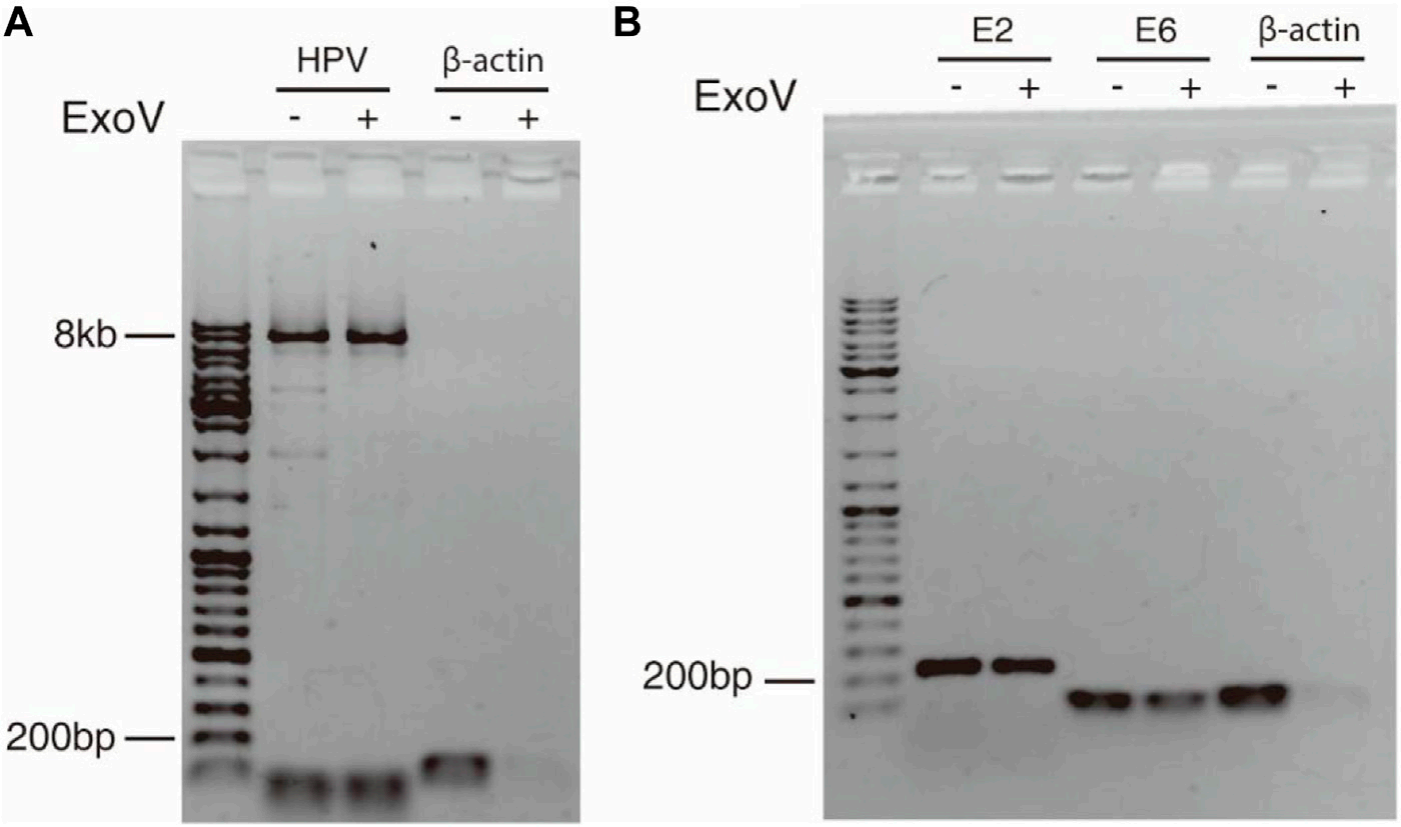
Circular episomal HPV DNA was detected by exonuclease V digestion and PCR. **(A)** DNA extracted from cervical cancer tissue was digested with exonuclease V and the HPV genome was amplified by long-range PCR using a pair of divergent primers. **(B)** E2 and E6 were amplified with E2 and E6 primers from DNA treated with or without exonuclease V. β-actin served as a control.

The discovery of concatemeric genomic sequences raises a question on whether HPV genome in cancer tissue exists in the episomal or integrated form. Exonuclease V digestion and long-range PCR (Forslund et al., 2019) were applied to address this question. Because exonuclease V specifically digests linear DNA but not circular DNA, circular HPV genome can be amplified with a pair of divergent primers within L1 gene (Table 1) from DNA treated with exonuclease V. The circular form of HPV genome was detected in cervical cancer tissue as shown in Figure 4, suggesting that the 11.54 kb-long concatemeric HPV sequence revealed by nanopore sequencing could be an intermediate product during the rolling circle replication of HPV genome. To further determine the physical status of the HPV genome in the cancer tissue, we tested the resistance percentage of E2 and E6 to exonuclease V digestion, followed by qPCR (Myers et al., 2019; Myers et al., 2020). Results showed that the average resistance of E2 and E6 to exonuclease V was in the range of 0.2–0.4 (Figure 4; Table 2), while the average resistance of β-actin to exonuclease V was almost 0, which suggests that integrated and episomal HPV genome coexist in the cervical cancer tissue. In contrast, in CaSki cells, the resistance percentages of E2, E6, and β-actin to exonuclease V digestion were almost 0 (Table 2), suggesting that only integrated forms of HPV genome exist. This result is consistent with that of a previous study (Myers et al., 2019).

**TABLE 2:** Physical state determination of the HPV genome by exonuclease V (ExoV)-qPCR–based assay.

Items	HPV35 in cervical cancer			HPV16 in CaSki		
Genes	E2	E6	β-actin	E2	E6	β-actin
Resistance to ExoV digestion	0.25	0.34	0.003	0.0005	0.0006	0.001
Physical state of HPV	Integrated and episomal			Integrated		

Note: a resistance rate of the detected genes equal to 1 indicates the HPV, genome is exclusively episomal; a rate close to 0 indicates exclusively integrated; between 0–1, mixed.

## Identification and Verification of HPV Integration Sites in Human Genome

**FIGURE 5:**
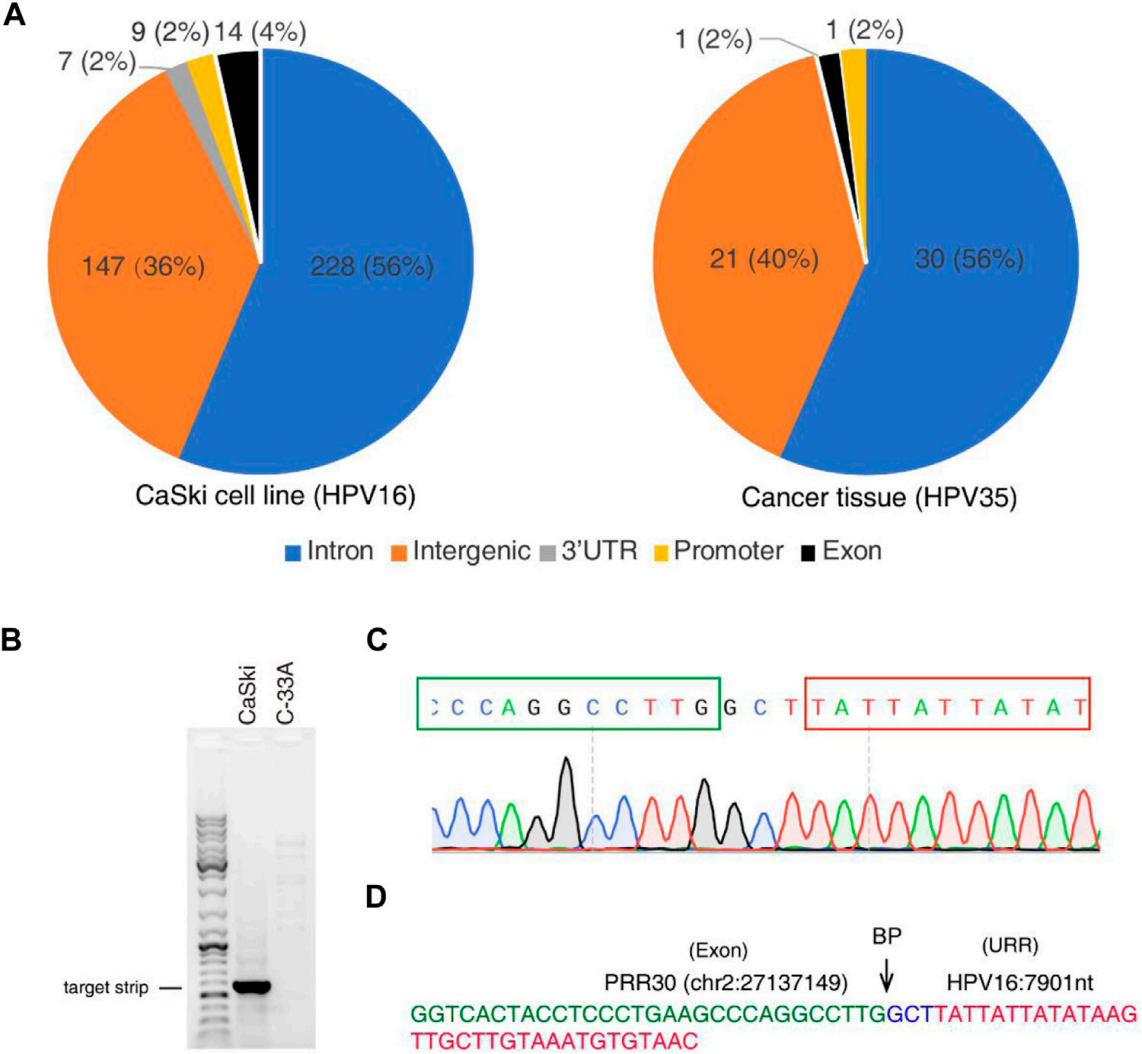
HPV integration sites identified by nanopore sequencing in CaSki cells and a cervical cancer tissue. **(A)** Distribution of HPV integration sites in different function regions of human genes identified from CaSki cells (HPV16) and a cervical cancer tissue (HPV35) by nanopore sequencing. **(B)** The HPV integration site was amplified by PCR from the CaSki cells, followed by agarose gel electrophoresis. C-33A cells (HPV-negative cervical cancer cells) was used as a negative control. **(C)** Sanger sequencing of the HPV integration site. PCR products were subjected to Sanger sequencing. Peaks of nucleotides at integration sites were shown. The cellular sequence from PRR30 was boxed with green color, and viral sequence was boxed with red color. **(D)** The sequence of the integration site located in PRR30 gene. The exon region of PRR30 gene was labeled with green color, and the URR region of HPV genome was labeled with red color. Three random nucleotides at the breakpoint (BP) were labeled with blue color.

To further identify HPV integration sites, a large number of closely located integration sites with distances less than 200 bp were considered the same, and 448 and 60 unique breakpoints were identified from the CaSki cell line and the cervical cancer tissue sample **(** Supplementary Tables S1, S2). HPV integration sites are mainly distributed in introns and intergenic regions as shown in Figure 5A, which is consistent with the results of previous studies (Koneva et al., 2018; Pinatti et al., 2018). Very few integration sites are located in exon, promoter, and 3′-UTR regions. For example, an integrated site at the exonic region of PRR30 in CaSki cells was revealed by 4 reads in nanopore sequencing data. To further verify this integration site, the integration region was amplified with PCR primers listed in Table 1, and PCR products (Figure 5B) were further sequenced by Sanger sequencing (Figures 5C,D). The HPV integration located in this exon is likely to affect the transcription and translation of PRR30, and further investigation is warranted.

## Discussion

Nanopore sequencing technology has attracted enormous attention since its release by Oxford Nanopore Technologies in 2014 due to its unique capability of generating ultra-long reads compared with secondary generation sequencing platforms. Nanopore sequencing has been applied for whole genome sequencing for humans (Jain et al., 2018), bacteria (Moss et al., 2020), and viruses (Beaulaurier et al., 2020). However, the error rate of nanopore sequencing remains a big concern. In this study, by sequencing cervical cancer tissue with nanopore sequencing, 10 x coverage of human genome and 97-fold coverage of the HPV genome were achieved; a *de novo* assembled HPV 35 genome is 99.96% identical to HPV gnome assembled by Sanger sequencing. In contrast, for CaSki cells, with 10 x coverage of human genome and 3,857 x coverage of HPV genome, the assembled HPV 16 genome is 99.99% identical to previously published reference genome. Although the low abundance of HPV positive cells in the cervical cancer tissue sample resulted in much lower coverage of HPV genome compared to CaSki cells, whole genome sequencing by nanopore sequencing can still generate HPV genome with high accuracy using our improved bioinformatic strategy.

Nanopore sequencing also demonstrated its advantage in detecting the physical status of the HPV genome in cervical cancer tissues. The level of HPV integration is positively correlated with the cervical intraepithelial neoplasia (CIN) grade and progression stages (Andersson et al., 2005; Briolat et al., 2007). Thus, identifying the episomal and integrated status of the HPV genome in cervical cancer will yield insight into HPV-induced cervical carcinogenesis. During viral replication, episomal circular genomic DNA co-exists with concatemeric amplicons as HPV adopts a rolling circle mode of replication (Flores and Lambert, 1997). Integration events presumably occur following the breakup of circular genomes or concatemeric amplicons under certain circumstances. Integration of a single genome or concatemeric amplicon is referred to as type I or type II integration, respectively (Baker et al., 1987; McBride and Warburton, 2017). The integration mechanism is poorly understood. Through nanopore sequencing, we not only found integrated sites with high efficacy compared to the NGS platform (Hu et al., 2015) but also found different integrated patterns of HPV genome exhibited in long reads as shown in Figure 3. This unique feature will yield insight into the integration mechanism of HPV in cervical cancer cells and facilitate our understanding of the role of HPV in carcinogenesis.

Previous works have found that HPV integration is not a random event, preferring to specific chromosomal regions, including oncogenes or tumor suppressor genes (e.g., BCL2, FANCC, HDAC2, RAD51B, and CSMD1) (Rusan et al., 2015; Groves and Coleman, 2018). Our study also revealed that one integration site (chr8:4915392-HPV35:6168) in the cervical cancer tissue sample is located in a previously identified tumor suppressor gene CSMD1 (Escudero-Esparza et al., 2016). Besides, when compared with known cancer genes in the COSMIC database (Tate et al., 2019), twenty-nine integration sites overlapped with records in COSMIC. For example, CSMD3 and ZFHX3 are involved in ovarian cancer and endometrial carcinoma, respectively, which indicated that integration of HPV may lead to malfunctions of host oncogenes and tumor suppressors.

Currently, nanopore sequencing technology is widely used for sequencing DNA and RNA and determining the methylation status of DNA (Ni et al., 2019) and RNA (Lorenz et al., 2020). Our study demonstrated that the nanopore sequencing platform is a unique tool to assemble HPV genome and study the integration mechanism. Long reads simplify the genomic assembly algorithm and further improve the accuracy of genomic assembly and integration analysis.

## Data Availability

The datasets presented in this study can be found in online repositories. The names of the repository/repositories and accession number(s) can be found below: https://ngdc.cncb.ac.cn/search/, PRJCA006562; HRA001318, HRA001319.
